# RutiSafeNet: a behavioral risk and nursing workload monitoring tool for open-door acute inpatient mental health units

**DOI:** 10.3389/fpsyt.2026.1882340

**Published:** 2026-07-28

**Authors:** Laura Miro-Mezquita, Anna Moreno-Orea, Joan de Pablo-Rabasso, Maria Isabel Martinez-Segura, Marina Delgado-Mari, Sandra Castro-Anso, Maria Jimenez-Murcia, Ana Ibañez-Caparros, Tatiana Bustos-Cardona, Jorge Cuevas-Esteban

**Affiliations:** 1 Servei de Psiquiatria, Hospital Germans Trias i Pujol, Badalona, Spain; 2 INnovation, health Economics and DIgital Transformation (INEDIT) Research Group on Innovation, Health Economics and Digital Transformation, Institut de Recerca Germans Trias i Pujol, Badalona, Spain; 3 Directorate of Healthcare Strategy and Innovation, Hospital Universitari Germans Trias i Pujol, Badalona, Spain; 4 NURECARE-IGTP Nursing Research Group, Institut Germans Trias i Pujol, Badalona, Spain; 5 Institut de Recerca Germans Trias i Pujol, Badalona, Spain; 6 Departament Psiquiatria, Universitat Autonoma de Barcelona, Barcelona, Spain; 7 Centro de Investigacion Biomedica en Red de Salud Mental, Madrid, Spain

**Keywords:** acute psychiatry, behavioral risk, inpatient mental health, nursing workload, open-door policy, patient safety, qualitative research, risk assessment

## Abstract

**Introduction:**

Open-door policies for acute inpatient mental health units (AIMHU) have shown promising results in reducing coercive measures, but concerns remain among patients and staff regarding increased workload, constant surveillance, and potential safety risks associated with these policies. This study aims to develop a monitoring tool to facilitate safety management in AIMHUs by monitoring behavioral risks and nursing workload.

**Methods:**

This study employed a qualitative approach using the content analysis method. Data were collected through three in-depth interviews and two focus groups involving staff (n=19) from the AIMHU of the hospital.

**Results:**

34 items were identified to define behavioral risks related to self-harm and suicide, aggressiveness, and absconding, alongside factors affecting nursing workload. These items were categorized into three levels of risk: low, moderate, and high.

**Discussion:**

RutiSafeNet is a preliminary, observation-based monitoring prototype intended to support, rather than to predict, structured risk and workload monitoring in acute inpatient mental health units. As a qualitatively developed instrument, it requires psychometric validation, including inter-rater reliability, construct and criterion validity, predictive value, and clinical feasibility, before it can be implemented as a validated scale in clinical practice.

## 1 Introduction

Human rights have emerged as a central focus in the evaluation of mental health policies and strategies (1, 2) with international and national patient safety recommendations emphasizing the need to safeguard the rights and safety of individuals hospitalized with mental disorders (3). Acute inpatient mental health units often experience a high frequency of conflict-related incidents such as aggression, violence, or absconding. The structure of psychiatric inpatient wards, significantly influences treatment outcomes and patient experience (4). While traditional closed-door policies are designed to enhance safety and security, they are often perceived by both patients (5) and staff (6) as restrictive and non-therapeutic environments, contributing to emotional distress, hinder recovery and lead to an increased use of coercive measures (7, 8). In response to this issue, different research initiatives have focused on implementing open-door policies for acute mental health hospitalization, showing promising results in reducing coercive therapeutic measures (9–11). Recent evidence suggests that open-door policies in psychiatric settings can reduce the use of coercive measures without increasing incidents of violence or harm, supporting their potential as a less restrictive and effective approach to inpatient care (12, 13).

Qualitative studies involving both patients and staff reveal differing perspectives on open-door policies in AIMHUs (14–18). Patients generally perceive these policies as supportive and therapeutic, enhancing safety, autonomy, and social interaction (6, 13, 19). However, some report discomfort with constant observation, highlighting tensions between safety and personal freedom (14, 20). Staff acknowledge the reduction in coercion but note increased workload and the need for continuous risk assessment to maintain control (21). Despite recognizing the benefits, concerns remain regarding heightened responsibility, stress, and potential safety risks (19, 22–24). Overall, these findings stress the need for reliable tools to safely implement open-door models and effectively manage unpredictable clinical situations.

To predict conflictive behaviors in mental health units, it is essential to identify the behavioral risk factors that trigger such events. Numerous studies have investigated these risks in depth. For instance, Powell et al. (2000) identified suicide-related risk factors in hospitalized patients, including suicidal ideation, history of self-harm, recent bereavement, presence of delusions, chronic mental illness, and family history of suicide (25). To assess the day-to-day risk of imminent aggression, the Dynamic Appraisal of Situational Aggression – Inpatient Version (DASA-IV) was developed based on seven behavioral indicators such as irritability, impulsivity, and verbal threats (26). Similarly, Georgieva et al. (2012) proposed a predictive model for the use of coercive measures like seclusion, incorporating variables such as involuntary admission, psychological impairment, and uncooperativeness (27). In terms of absconding risk, the Waypoint Elopement Risk Scales (WERS) assess both clinical history (e.g., previous absconding attempts, substance abuse, self-harm) and recent events (e.g., threats to abscond, life stressors, treatment adherence) (28).

In addition to behavioral risks, it is crucial to monitor the nursing workload associated with each patient according to their clinical needs. This workload contributes to the overall risk assessment and informs decisions regarding the feasibility of open-door policies (29). However, current tools for evaluating nursing workload in psychiatric settings are scarce and lack standardization. A scoping review by Sousa & Seabra (2018) identified only four relevant studies (29–32). Among them, Petrucci et al. developed a complexity indicator based on 88 items aligned with Gordon’s 11 functional health patterns (33). Other studies focused on workforce planning but did not enable the continuous monitoring of workload variations throughout the hospital stay. More recent instruments also rely heavily on staff hours (34, 35), further limiting their applicability for real-time decision-making.

In the context of psychiatric hospitalization, particularly under open-door policies, it is vital to continuously assess both behavioral risks and nursing workload to ensure the safety of patients and staff. To our knowledge, no existing instrument offers an objective, observation-based assessment of both domains simultaneously and throughout the entire course of hospitalization. Therefore, this study aimed to develop a prelimiray structured, observation-based monitoring tool to assess behavioral risk and nursing workload in an open-door acute inpatient mental health unit. The tool was designed not only to categorize individual patient risk, but also to support shift-level clinical decision-making regarding safety management, staff prioritization, supervision needs, and the feasibility of maintaining open-door conditions. It was conceived as a monitoring and decision-support aid rather than as an individual-level risk-prediction algorithm.

## 2 Methods

### 2.1 Study design

The aim of this study is to design a monitoring tool for inpatients that allows for the categorization and quantification of behavioral risk and nursing workload levels to support ward-level safety management and provide an objective measure to inform open-door decisions. To address the objective of this study, a qualitative investigation was undertaken through individual interviews and focus groups during the first quarter of 2023. Before initiating the qualitative study, the interviewer, that is the first researcher, conducted an observational analysis of the unit to manage interview time effectively, prioritize topics based on their importance, and prevent participant dispersion.

An expert panel, composed of psychiatrists, a nurse manager and senior mental health nurses, initially defined the two main categories, behavioral risks and nursing workload. Based on the literature, it was also considered relevant to subdivide the behavioral risk category into subcategories: self-harm and suicide, aggression, and absconding. The tool is designed to monitor each patient in both main categories, behavioral risk and nursing workload, throughout the entire hospital stay. To classify the levels of behavioral risk and nursing workload, the expert committee defined three levels as a starting point: low, moderate, and high, associated with values of 0, 1, and 2, respectively.

Each item was assigned a severity score corresponding to a moderate (1) or high (2) level, together with a predefined duration for which the item remains active once observed (e.g., one shift, three shifts, or the entire admission). At any given moment, a patient’s score in each main category is defined as the highest score among the items currently active in that category. Accordingly, a category score of 0 (low) denotes the absence of any active moderate- or high-level item; a score of 1 (moderate) denotes that the highest active item is of moderate level; and a score of 2 (high) denotes that at least one high-level item is active. The three behavioral-risk subcategories (self-harm and suicide, aggression, and absconding) are scored independently using the same rule, and the behavioral-risk category score corresponds to the highest of the three subcategory scores. The two main categories were given equal weight as an *a priori*, pragmatic decision by the expert panel, taken in the absence of empirical data on their relative contribution to ward safety and intended to keep the instrument transparent and simple at this developmental stage; the empirical estimation of differential weights is an explicit objective of future validation. At ward level, the individual category scores of all admitted patients are summed to yield an aggregate indicator intended to reflect the overall safety climate of the unit and to inform shift-level decisions, including the feasibility of maintaining open-door conditions. The decision thresholds derived from this aggregate are considered preliminary and require prospective empirical validation.

### 2.2 Setting

The present study was conducted at the Acute Inpatient Mental Health Unit of Germans Trias University Hospital (GTUH), Badalona, Barcelona, Spain, a general multispecialty hospital affiliated to the University Autonoma of Barcelona. At the time of the study, GTUH catchment area was of 350530 adults distributed across four community mental health areas in the North Barcelona health district, with a mean of 313 adult discharges per year. In Spain, involuntary psychiatric admission is regulated by Article 763 of the Civil Procedure Act (Ley 1/2000 de Enjuiciamiento Civil), which requires judicial authorization of the measure; this legal status is clinically relevant to the present tool, as the type of admission modulates several absconding-related items. The acute inpatient mental health unit at GTUH consists of one 20-bed ward and is staffed by multidisciplinary teams of senior and junior psychiatrists, nurses, psychologists, and social workers. The unit provides multidisciplinary acute specialist clinical care for patients admitted with serious mental illnesses, in the form of pharmacotherapy, psychotherapy, social work and occupational care, and a recovery program. Since 2019, the unit has progressively implemented the Safewards care model (36). Safewards is a set of ten interventions designed to enhance safety by preventing conflict and containment. Electroconvulsive therapy is available when the study was carried out. Most of the patients (90-95%) are admitted from the emergency room. They usually suffer from severe primary psychiatric disorders with acute disturbances or risk of self-harm. The remaining of patients are referred from community mental health providers. The ward operates under an open-door policy, with doors open during daytime and closed at night. This policy has been implemented since October 2021.

### 2.3 Reflexivity

The self-reflexive knowledge of the researchers in this study is as follows: The first researcher (LMM) is a Biomedical Engineer (MSc) that has actively participated in methodological issues related to hospitalization processes. The fourth researcher (MIMS, MSc) possesses experience in qualitative research and has knowledge of how the unit operates. The fact that the researchers conducting the interviews, and the focus groups were not directly involved in daily clinical practice facilitated participants’ free expression. The second researcher (AMO) is a nursing manager (MSc) of the unit; the third (JCE) and the last researcher (JdPR) are the psychiatrists (Ph.D) responsible for implementing open-door policies in the GTUPH’s mental health unit. Although AMO holds a managerial role, the interviews and focus groups were conducted by researchers who were not part of the participants’ clinical line management (LMM and MIMS), and reflexive team discussions were held throughout the analysis to limit the potential influence of these professional relationships on data collection and interpretation.

### 2.4 Rigor

This research was conducted according to the guidelines in the Standards for Reporting Qualitative Research (SRQR) (37). To establish credibility, independent coding of the data was done by two researchers (LMM and MIMS), followed by a collaborative consensus on emerging codes. Transferability was addressed by providing detailed descriptions of the AIMHU setting and the open-door policy context, allowing readers to assess the applicability of findings to their own environments. To ensure dependability, we have clearly outlined the data collection and analysis processes, including the interviews and focus groups methodologies. The study adhered to ethical guidelines, receiving approval from the local research ethics committee (PI-23-143).

### 2.5 Participants

The inclusion criteria for participants were as follows: (1) be working in the GTUH ‘s acute mental health unit, and (2) having experience, at least six months, working on open-door policies. Participants were selected through purposive (purposeful) sampling, a non-probabilistic strategy in which information-rich individuals are deliberately selected because of their direct experience with the phenomenon under study (38, 39). We sought maximum variation across professional roles (psychiatrists, nurses, nursing assistants, a psychologist and a social worker) and across levels of seniority, so that the full range of clinically relevant perspectives on behavioral risk and nursing workload would be represented, rather than a statistically representative sample. The participants were selected using the purposive method. The individual interviews included 3 participants, two females, a psychiatrist and a nurse, and a male as nursing assistant, to be as representative as possible of the different areas of work. They were contacted face-to-face during their work shifts in the mental health unit and explained both the objectives and the procedure to be followed, and everyone accepted. For the focus groups, a total of 29 participants were contacted by email and 16 agreed to take part in the study. The response rate was 55.17%. All the participants received detailed information about the purpose and scope of the study by email or in a preliminary interview, and informed consent was obtained from all subjects involved in the study.

For the individual interviews, three professionals were approached face-to-face during their shifts. We acknowledge that approaching staff within the workplace may raise concerns regarding voluntariness and perceived pressure to participate, as well as practical constraints on the time available for reflection. To mitigate these risks, recruitment and data collection were carried out by researchers (LMM and MIMS) who were not part of the participants’ clinical line management and had no role in their performance appraisal; participation was explicitly framed as voluntary and refusal as inconsequential; interviews were scheduled so as not to interfere with patient care; and written informed consent was obtained after a full explanation of the study. Focus-group participants were instead invited by email, which allowed time to consider participation without immediate workplace pressure. We further recognize that workplace recruitment and the presence of a nursing manager within the research team could introduce social-desirability effects; reflexive team discussion and independent double coding were used to limit their influence on the analysis.

Of the 29 professionals invited to the focus groups, 16 agreed to participate (approximately 55%). Although this rate is acceptable for staff-based qualitative research, we acknowledge the possibility of volunteer bias, whereby professionals with stronger views on, or greater interest in, open-door policies may have been more likely to take part. Because the study followed a purposive, saturation-driven logic rather than aiming for statistical representativeness, this does not undermine its qualitative objectives; nevertheless, the findings should be interpreted as reflecting the perspectives of an engaged subset of the unit’s staff.

Two focus groups were conducted, with 8 participants per session. The participants included 8 nurses, 5 nursing assistants, 1 psychiatrist, 1 psychologist and 1 social worker. Of the participants, 81.25% were females, while the rest were males. The focus groups were held at the GTUH facilities. The sociodemographic data for all the participants is shown in **Table 1**.

**Table 1:** Descriptive statistics of participants in the qualitative study.

Variable	mean (years)	SD (years)
Age	36.61	10.83
Work experience	11.33	8.98
Experience at GTUH	8.09	8.68

### 2.6 In-depth semi-structured interviews

The data were collected through face-to-face semi-structured interviews conducted by a researcher (LMM) in a designated room within the AIMHU of the GTUH. Only the researcher and the interviewee were present in the room. Prior to the interviews, an interview guide was developed by the expert panel containing standardized questions for all three interviewees, along with tailored elaborations based on everyone’s profession.

Initially, the interview began by asking which approach was considered more equitable for evaluation: whether consolidating the main categories into a single measure or separating behavioral risk into subcategories such as absconding, self-harm and suicide, and aggression was preferable, and why. Hereafter, in accordance with the methodology of qualitative studies, the term “categories” will refer to themes, and “subcategories” will denote subthemes. The objective was to validate behavioral risks subcategories. After this discussion, each interview was structured into two sections according to the categories: behavioral risks and nursing workload. Behavioral risk section was subdivided in the three different subcategories. Each section started with the following question: “Do you think it is appropriate to differentiate it into low, moderate and high level?”. It was followed by an open-ended query depending on the subtheme addressed: “What observations would you employ as criteria to delineate whether a patient is categorized as low, moderate, or high risk?”. These initial prompts were succeeded by further exploratory inquiries for each behavioral risk, such as: “What behavioral characteristics are exhibited by patients at risk of aggression?” “What common traits do patients who attempt to abscond share?”.

To objectively assess nursing workload, the inquiries included the same initial questions for the levels of categorization and initial items. Moreover, workload disparities between voluntary and involuntary inpatients were discussed and identifying physical care needs that require increased staff attention was a crucial issue. The influence of the mental status in the nursing workload was also explored. The unstructured nature of the questions facilitated participants in freely recounting their experiences. Detailed field notes were taken during the interviews.

The duration of the interviews averaged approximately 45 minutes for each participant. They were audio-recorded using two distinct devices, with participants providing verbal consent prior to commencement. Subsequently, the interviews were meticulously transcribed in accordance with extended transcription guidelines tailored for scientific discourse, encompassing verbatim transcription with minor language corrections and adaptation of dialects to adhere to the Spanish standard language. All transcriptions underwent thorough scrutiny and proofreading by research assistants.

Data analysis followed the qualitative content analysis method described by Elo and Kyngäs (40), combining a deductive framework, the two main categories (behavioral risk and nursing workload) and the three behavioral-risk subcategories were defined *a priori* by the expert panel, with the inductive identification of items within each category. Two researchers (LMM and MIMS) independently performed line-by-line coding of the verbatim transcripts and field notes, labelling meaning units as descriptive codes (referred to as “items” throughout the article and in the developed tool) and provisionally assigning each item to a category and a severity level. Coding was carried out manually, without computer-assisted qualitative data-analysis software. The two coders subsequently compared their independent coding and resolved discrepancies through discussion until consensus was reached; conceptually overlapping or redundant items were merged and ambiguous items reworded. The expert panel intervened at two clearly delimited points: *a priori*, to define the categorical framework, and a posteriori (after the focus groups), to consolidate the data-derived items, confirm the severity level and alert duration of each item, and add items judged clinically essential that had not been spontaneously raised. Item generation itself remained grounded in the qualitative data. Participant quotations were translated into English by the research team for publication.

### 2.7 Focus groups

Two focus groups were conducted in a hospital training room, guided by two researchers. Based on insights extracted from the analysis of interview data, a comprehensive guide was developed to direct the focus groups toward relevant topics, especially those generating the most discussion. No additional individuals were present in the interview room aside from the participants and the researchers. Each session adhered to a structured format comprising an agenda overview, contextualization, presentation of objectives, and subsequent thematic and sub-thematic discussions. During these discussions, items and the corresponding levels (low, moderate and high) extracted from individual interviews were first introduced and deliberated upon. After that, other situations were exposed while researchers diligently documented field notes and identify new items as necessary. Participants were guided by the researchers to remain on topic. It was considered highly relevant to collect information on the appropriate duration of alert maintenance for each item. Each focus group extended over a duration of 1.5 hours, and data saturation was achieved in both instances. As in the interviews, the recordings were made with two different devices and transcribed after the session. Before starting, the participants gave their consent. The analysis of the information mirrored the content analysis methodology used in the interviews; the study data were categorized by two researchers and were coded independently. After the interviews and focus groups, coding was compared and categorized according to similarities and differences. Finally, it was decided to convene the expert panel to refine, consolidate, and validate the items for each level to the greatest extent feasible, respecting the results obtained from the focus groups. Data saturation was considered achieved when no substantially new items or dimensions emerged during the second focus group and the expert panel confirmed that the proposed categories covered the relevant risk and workload domains. The adequacy of the sample was therefore judged on the basis of information power and data saturation rather than numerical size (41), consistent with the purposive design and the diversity of professional roles represented.

## 3 Results

The result of this study is a tool for monitoring acute patients in mental health units during admission, which will be called RutiSafeNet. In the qualitative study that was carried out to develop it, categories were previously defined, and subcategories were validated.

### 3.1 Behavioral risks

First, the debate revolved around which types of situations should be included in the tool. Collectively, they agreed that, since the tool aims to monitor changes during hospitalization, it should focus more on clinical presentation and individual behaviors rather than clinical history and intrinsic patient information.

“I believe it’s more about the clinical presentation than the patient.” (P9)

“Yes, because the behavior can vary greatly.” (P10)

Regarding self-harm and suicide risk, all participants unanimously agreed that constant verbalization of suicidal ideation poses a high risk, while a punctual verbalization of self-harm wishes represented a moderate risk. Any type of self-injury or medication overdose was also agreed upon to merit a high score.

“And a high risk, well, that it comes from a well-structured suicidal thought, or because it has been carried out or is manifesting that it is going to do it already without telling you how. Also, that directly during the shift it is evidenced that self-injury or medication overdose has been performed.” (P1)

“The behaviors that increase our risk are autolytic threats, failure to criticize an autolytic attempt, engaging in any activity while in the unit, attempting to access medication, or attempting self-harm at any time…” (P3)

Ten items were ultimately retained in the self-harm and suicide subcategory. In addition to the high-risk items already described, constant verbalization of suicidal ideation, moderate or severe self-harm, and suicidal attempts, participants classified as high risk the act of hiding an object with self- or other-harm potential, the stockpiling of medication, and psychomotor pre-agitation, all of which were understood as signaling an imminent capacity or preparation for harm. Several indicators were instead classified as moderate risk: the verbalization of passive death wishes, mild self-harm occurring during the shift, suspicions of suicidal risk expressed by the family, and hiding in blind spots of the unit’s camera coverage. A recurrent theme was the differentiation of suicidal thinking by its intensity and structure, with participants reserving the high-risk level for structured or enacted ideation and assigning the moderate level to passive or fleeting expressions.

Picking up dangerous objects such as sharp objects, lighters, and belts was identified as posing a high risk of aggressiveness to both themselves and others. Additionally, there was discussion about the severity of verbal threats, with the minimum score being moderate risk.

“When in doubt, I would always mark it as red because you never know what could happen, but the type of verbal threat should be considered.” (P2)

Five items were retained in the aggressiveness subcategory. Realized aggressive behavior, whether directed toward people or toward objects, and the situation of a recently unrestrained patient were classified as high risk, reflecting both the immediate hazard and, in the case of recent restraint, the elevated short-term probability of renewed agitation that warrants continued close observation. Verbal aggressiveness and threatening behavior were classified as moderate risk; participants noted that the severity and credibility of a threat should inform the level assigned, while advising a precautionary approach when in doubt. The discussion on access to potentially dangerous objects was ultimately operationalized under the self-harm and suicide subcategory (“hiding an object with potential for self/hetero harm”), given its dual relevance to self- and other-directed harm.

Regarding absconding risk, hypervigilance with access points and requests for personal documentation were mentioned as indicators of considerable risk. Previous absconding attempts were considered high risk in involuntary inpatients.

“If they come in very decompensated and have a lot of history of absconding, it’s more likely that they will leave than if they come in for a change in treatment or because they are awaiting another device.” (P6)

Eight items were identified within the absconding subcategory. Participants distinguished overt, behaviorally explicit indicators, classified as high risk from less specific or contextual indicators, classified as moderate risk. Among the high-risk items, an explicit verbal manifestation of the intention to leave and hypervigilance with access points (for example, repeatedly checking or lingering near doors and exits, or requesting personal documentation) were considered the clearest behavioral signals of imminent absconding. Previous absconding attempts were also regarded as high risk, but only in involuntarily admitted patients, as participants emphasized that the clinical meaning of a prior attempt depends on the patient’s legal admission status, as illustrated in the quotation above (P6). The moderate-risk items captured situations that increase the likelihood of absconding without constituting a direct behavioral signal: constant demands for discharge, refusal to take medication, an acute cognitive change, moderate-to-severe cognitive impairment with preserved ambulation (which may lead to unintentional wandering through open doors), and involuntary admission itself. For these contextual items, participants stressed that the level of alert should be modulated by admission status and by the patient’s overall clinical state rather than treated as a fixed attribute.

### 3.2 Nursing workload

The definition of nursing workload score revolved around the cognitive status of patients and their demands.

“Very high is a person who is all the time in the control room, asking for our attention, that you have to be with him during the shift, that is very invasive, very reiterative.” (P5)

“Medium level, would be a depression for example that needs us to be there doing reconductions.” (P1)

Patients requiring moderate demands such as assistance with hygiene or meal companionship were deemed to impose a moderate workload. Patients with a high degree of dependence and those requiring constant redirection were identified as posing the highest burden, warranting a score of two.

Twelve items were retained in the nursing workload category, which participants organized around three sources of demand: dependency for basic care, the need for supervision or accompaniment, and additional clinical or organizational demands. High-workload (score 2) items included dependency for activities of daily living, the need for constant accompaniment, mechanical or environmental restraint, and isolation due to a contagious condition. Moderate-workload (score 1) items included a demanding patient, the need for assistance with hygiene, intermittent accompaniment, the presence of an eating disorder, the reversal of oppositional behavior, and the need for nursing care for an acute somatic pathology. Participants underscored that workload was the most debated category, reflecting both its subjectivity and its direct impact on staff, and that the most burdensome profiles combined a high degree of dependence with a continuous need for redirection and supervision.

In an initial review led by the first and fifth researchers, 59 items and their corresponding scores were extracted from the in-depth interviews and focus groups. Following an analysis of conceptual similarities and regrouping efforts, 31 items along with their corresponding scores were delineated. Following the review session conducted by the expert panel, the qualitative analysis was finished. The duration for maintaining the alert status for each item was determined. Finally, **Table 2** presents the 34 items and their corresponding score and duration for categorizing risks associated with self-harm and suicide, aggressiveness, and absconding, as well as the nursing workload attributed to patients.

**Table 2:** Items, score and duration for self-harm and suicide, aggressiveness and absconding risk and nursing workload extracted from the complete qualitative study.

	Items	Score (level)	Duration
Behavioral risks			
Self-harm and suicide risk			
	Verbalization of suicidal ideation	2	Three shifts
	Verbalization of passive death wishes	1	One shift
	Moderate/severe self-harm incidents	2	Three shifts
	Mild self-harm during shift	1	One shift
	Suicidal attempts	2	Three shifts
	Hiding an object with potential for self/hetero harm	2	Three shifts
	Stockpiling medication	2	Three shifts
	Suspicions of suicidal risk expressed by the family	1	Three shifts
	Hiding in blind spots of the camera	1	One shift
	Psychomotor pre-agitation	2	Three shifts
Aggressiveness risk			
	Aggressive behavior toward people	2	Three shifts
	Aggressive behavior toward objects	2	Three shifts
	Verbal aggressiveness	1	One shift
	Threatening behavior	1	Three shifts
	Recently unrestrained patient	2	Three shifts
Absconding risk			
	Explicit verbal manifestation	2	Three shifts
	Hypervigilance with access points	2	Three shifts
	Constant demands for discharge	1	One shift
	Refusal to take medication	1	One shift
	Acute cognitive change	1	One shift
	Previous absconding attempts in involuntarily admitted patients	2	Entire admission
	Moderate/severe cognitive impairment with ambulation	1	Entire admission
	Involuntary admission patient	1	Until voluntary admission
Nursing workload			
	Demanding patient	1	One shift
	Patient dependent for activities of daily living	2	One shift
	Patient requiring assistance with hygiene	1	One shift
	Patient in environmental restraint	2	One shift
	Isolation due to contagious condition	2	One shift
	Patient with eating disorder	1	One shift
	Patient in mechanical restraint	2	One shift
	Requires constant accompaniment	2	One shift
	Requires intermittent accompaniment	1	One shift
	Requires nursing care for acute somatic pathology	1	One shift
	Reversal of oppositional behavior	1	One shift

0: low risk/workload (absence of any active moderate or high-level item in the subcategory).

1: moderate risk/workload.

2: high risk/workload.

Each item, once observed, remains active for the indicated duration; a patient’s score in each subcategory corresponds to the highest score among the items active at a given time.

## 4 Discussion

This study presents the development of a preliminary structured monitoring tool for managing patient risk in mental health hospitalization. The prototype is based on 34 items, differentiated into two main categories, behavioral risks and nursing workload and specifically assesses self-harm and suicide, aggression and absconding risk. The categories and subcategories were considered appropriate in the study, aligning with the risk division defined by the National Health Service (2006) (3). The distinction into three levels was also regarded as suitable for each category and subcategory.

It should be emphasized that RutiSafeNet was conceived as a monitoring and decision-support instrument rather than as a risk-prediction algorithm. Accordingly, the item set deliberately combines two types of indicators: dynamic warning signs that may precede an adverse event (for example, psychomotor pre-agitation, hypervigilance with access points, or the verbalization of suicidal ideation) and recent realized incidents (for example, a suicidal attempt, aggressive behavior toward people, or a recently unrestrained patient). In a monitoring framework, a realized incident is not used to predict the event that has already occurred; rather, it activates a time-limited state of heightened observation and increased nursing attention, reflecting the recognized short-term elevation of recurrence risk and supervision need that follows such events. This distinction between prospective warning indicators and incident-triggered observation states has been made explicit throughout the revised manuscript, and predictive terminology has been avoided.

Regarding self-harm and suicide risk, it was concluded that verbalization of suicidal ideation, moderate or severe self-harm, and suicide attempts address high risk, coinciding with the conclusions drawn by Powell et al. (25). Other factors that have been considered to represent high risk are hiding an object with self-harmful potential, accumulate medication and psychomotor pre-agitation. In the present study it has been reported that the severity of suicidal ideation can be differentiated into two risk levels depending on the severity. Passive death wishes and non-suicidal self-injury have been considered of moderate risk. Furthermore, events that are not intrinsic to the patient have not been taken into consideration, such as previous family history of suicide or recent bereavement because the tool is designed to be based on observational items.

Items of the aggressive category that score as high risk are aggressive behavior toward people, toward objects and recently unrestrained patient. Verbal aggressiveness and threatening behavior are considered medium risk. The DASA-IV (26) scale item 'Verbal threats' is directly related to verbal aggression, but the four remaining items extracted from this qualitative study do not have an equivalence in the DASA-IV scale.

In respect to absconding risk subcategory items, explicit verbal expression was considered a high risk, as also defined in the WERS scale (28). Hypervigilance with access points, which has been concluded to pose a high risk of absconding, is not included in the WERS scale. On the other hand, within this subcategory, it has been established that previous absconding attempts only represent a risk in patients with involuntary admissions, whereas the WERS scale considers them regardless of the type of admission. For this item, the type of admission was considered to assign a confidence rating to the patient, as previous attempts to abscond may have occurred a long time ago or could be related to other conditions. Although the WERS scale does not address this issue, Georgieva et al. (27) reported that it had high predictive power for seclusion and the use of coercive measures. The medium risk item ‘refusal to take medication’ is also included in the WERS scale, while ‘acute cognitive change’ can be reflected in the WERS item ‘behaviors.’ The remaining items from this qualitative study that indicate a medium risk of absconding, namely, constant demands for discharge, moderate to severe cognitive impairment with ambulation, and involuntary admission, do not have equivalents in the aforementioned scale.

Finally, the nursing workload category includes items related to patient care, excluding those associated with administrative tasks. This category generated the greatest discussion among participants, likely due to its inherent subjectivity and its significance to staff, as opposed to behavioral risks. In this study, different levels of workload have been distinguished, while in the tool developed by Petrucci et al. (29), all items receive the same score. One of the major differences between this tool and the scale developed by Petrucci is that the latter only considers the nursing workload at the time of admission, whereas ours assesses it during each shift. This enables the acquisition of current information regarding patient needs and the associated workload. In the "health management" category of Petrucci's tool, acute somatic pathology care and management of patients with oppositional behaviors are included; in the "Activity/exercise" category, items related to dependency on basic activities of daily living are considered; and in "Nutritional," patients irregularly consuming food or liquids are scored, although it may not be related directly to an eating disorder. The oppositional behaviors are also included in the DASA-IV scale. Some situations covered in the 88-item tool are also found in the one extracted from this qualitative study; however, they are not included in the nursing workload category, such as hypervigilance, psychomotor pre-agitation, and suicidal ideation. Items related to supervision (constant and intermittent accompaniment and demanding patient) are not considered in the Petrucci’s tool. The remaining items, patients in mechanical or environmental restraint, or with a contagious condition, have been classified in different levels in the nursing workload category.

This tool directly addresses the safety concerns raised by staff and patients regarding open-door policies, as highlighted in several qualitative studies (13, 14, 19, 23). Since the tool provides numerical values, individual scores can be aggregated to generate a ward-level indicator reflecting the overall safety climate of the unit. The aggregation of behavioral risk and nursing workload may offer insight into environmental safety and support decisions regarding the feasibility of maintaining open-door conditions. The three-level scoring system and the predefined duration of each alert were designed to transform qualitative clinical observations into actionable information for routine decision-making. In this sense, the tool provides not only an individual patient-level assessment, but also a structured output that may guide preventive actions at shift level, such as increasing observation, prioritizing staff attention, adjusting accompaniment needs, or reconsidering open-door conditions. The aggregation of individual scores may also facilitate the definition of decision thresholds for ward-level safety management. However, these thresholds should be considered preliminary and require future empirical validation to determine their reliability, predictive value, and clinical usefulness.

One of the strengths of the current study is that it was based on two focus groups, and 3 in-depth interviews with the collaboration of a wide variety of mental health professionals. Employing qualitative techniques like semi-structured interviews enables participants to elaborate on their answers, leading to a comprehensive data gathering process. To the best of our knowledge, it is the only study that simultaneously considers the behavioral risks of hospitalized patients and the workload they generate for the nursing staff. Moreover, due to the way the information in the tool is structured, organized into categories and subcategories, and the concise nature of the items, it is a user-friendly tool that requires minimal prior training.

This study has several limitations that define the developmental stage of the instrument. First and foremost, RutiSafeNet should be regarded as a preliminary prototype rather than a validated monitoring scale: it has been derived from qualitative evidence and expert consensus, but its psychometric properties have not yet been examined. Future research must therefore assess its inter-rater reliability, internal consistency, temporal stability, construct and criterion validity, predictive value, and clinical feasibility and acceptability before it can be recommended for routine use, ideally through prospective studies and comparison with established scales. Second, both the relative weighting of the two main categories and the alert durations assigned to each item rest on *a priori* expert consensus rather than on empirical data; equal weighting was adopted as a transparent and pragmatic starting point, and the durations reflect the clinical judgement expressed during the focus groups, but both parameters require prospective empirical calibration. Third, the study was conducted in a single 20-bed acute unit and drew exclusively on the perspectives of its own staff, which carries a risk of single-center and within-team bias and limits the transferability of the findings to units with different structures, casemix, or door policies. Relatedly, although a staff-operated, observation-based design is appropriate for an instrument whose explicit purpose is to support shift-level nursing and medical decisions, and although nursing workload is intrinsically a staff-defined construct, patients’ and informal caregivers’ perspectives were not incorporated; their inclusion in subsequent co-design and validation work would strengthen both the content validity and the acceptability of the tool. Fourth, the sample was recruited and, in the case of the interviews, partly approached within the workplace, which may have introduced volunteer and social-desirability effects, as discussed in section 3.5. Finally, in the nursing workload category, non-patient-related activities (such as training other staff and organizational or administrative work) were not considered, although their contribution to overall workload could be analyzed in future iterations. Lastly, member checking, returning the consolidated items to participants for verification, was not undertaken; future development could incorporate this step to further strengthen the credibility of the item set.

The findings suggest that risk in open-door inpatient units should be understood as a dynamic and relational construct. Safety depends not only on individual behavioral indicators, but also on contextual factors such as nursing workload, supervision needs, and the overall state of the ward. This supports the need for risk assessment approaches that account for complexity rather than relying exclusively on individual-level risk prediction.

## 5 Conclusions

This study underscores the importance of structured risk assessment in AIMHUs, particularly in the context of open-door policies. Through a qualitative, consensus-based process, we identified and categorized 34 items spanning behavioral risk and nursing workload and assembled them into RutiSafeNet, a preliminary, observation-based monitoring prototype intended to support, not to replace, clinical judgement in daily risk management. The proposed structure offers a transparent and potentially user-friendly approach to flagging self-harm, aggressiveness and absconding risk and to tracking nursing workload at shift level, with the aim of supporting clinical decision-making and patient safety. The instrument has not yet been validated: further research is required to establish its reliability, validity, predictive value, feasibility, and applicability across diverse settings, as well as its eventual impact on the use of coercive measures. Pending such validation, RutiSafeNet is best understood as a structured framework to be tested empirically rather than as a ready-to-use clinical scale; if confirmed, the standardization of risk and workload monitoring it proposes could represent a meaningful contribution to the management of AIMHUs, with the potential for broader implementation.

## Data Availability

The raw data supporting the conclusions of this article will be made available by the authors, without undue reservation.
